# Discovery of the Potential Novel Pharmacodynamic Substances From Zhi-Zi-Hou-Po Decoction Based on the Concept of Co-Decoction Reaction and Analysis Strategy

**DOI:** 10.3389/fphar.2021.830558

**Published:** 2022-01-13

**Authors:** Xin Feng, Yuelin Bi, Jiaqi Wang, Tianyi Li, Gengyuan Yu, Tonghua Zhang, Haoran Xu, Chenning Zhang, Yikun Sun

**Affiliations:** ^1^ School of Chinese Materia Medica, Beijing University of Chinese Medicine, Beijing, China; ^2^ Department of Pharmacy, Zigong First People’s Hospital, Zigong, China

**Keywords:** Zhi-Zi-Hou-Po decoction, co-decoction, UPLC-QE-Orbitrap-MS, new compound, molecular docking

## Abstract

**Background:** Zhi-Zi-Hou-Po Decoction (ZZHPD), a classic traditional Chinese medicine (TCM) formula, is clinically used to treat insomnia and depression. The analysis strategy based on the concept of co-decoction of TCM is helpful to analyse the effective substances of TCM formula in depth.

**Aim of the study:** This manuscript intends to take ZZHPD as a model sample to explore the phenomenon of co-decoction of complex formula in the combination of liquid chromatography-mass spectrometry (LC-MS) technology, data analysis, and molecular docking.

**Materials and methods:** In the current research, an innovative LC-MS method has been established to study the active ingredients in ZZHPD, and to identify the ingredients absorbed into the blood and brain tissues of mice. And molecular docking was used to study the binding pattern and affinities of known compounds of the brain tissue toward insomnia related proteins.

**Results:** Based on new processing methods and analysis strategies, 106 chemical components were identified in ZZHPD, including 28 blood components and 18 brain components. Then, by comparing the different compounds in the co-decoction and single decoction, it was surprisingly found that 125 new ingredients were produced during the co-decoction, 2 of which were absorbed into the blood and 1 of which was absorbed into brain tissue. Ultimately, molecular docking studies showed that 18 brain components of ZZHPD had favourable binding conformation and affinity with GABA, serotonin and melatonin receptors. The docking results of GABRA1 with naringenin and hesperidin, HCRTR1 with naringenin-7-O-glucoside, poncirenin and genipin 1-gentiobioside, and luteolin with SLC6A4, GLO1, MAOB and MTNR1A may clarify the mechanism of action of ZZHPD in treating insomnia and depression.

**Conclusion:** Our study may provide new ideas for further exploring the effective substances in ZZHPD.

## Introduction

Fast-paced city life has aggravated the pressure on people’s lives, causing people to fall into the dilemma of anxiety, insomnia and even depression, and prevalence of the symptoms has severely affected people’s quality of life. Long-term insomnia can induce many complications, and can even cause sudden death (Liu X et al., 2021). Zhi-Zi-Hou-Po Decoction (ZZHPD) is composed of three Chinese medicinal herbs: *Gardeniae Fructus* (GF), *Aurantii Fructus Immaturus* (AFI) and *Magnoliae Officinalis Cortex* (MOC) (Table 1)*.* ZZHPD is a classic herbal formula in traditional Chinese medicine (TCM), derived from “*Shang Han Lun*”, and has been used for the treatment of anxiety and insomnia for more than a thousand years (Xing et al., 2015). Scientists have revealed that the mechanism of ZZHPD in treating insomnia may be related to restoring the function of the hypothalamus-pituitary-adrenal axis, increasing the expression of hippocampal brain-derived neurotrophic factor and promoting the healing of the hippocampal nerve function (Zhu et al., 2020).

**TABLE 1:** Components of ZZHPD.

Plant names	Traditional Chinese medicine name	Abbreviations
Gardenia jasminoides J.Ellis	Gardeniae Fructus	GF
Citrus aurantium L	Aurantii Fructus Immaturus	AFI
Magnolia officinalis Rehder and E.H.Wilson	Magnoliae Officinalis Cortex	MOC

The controversy between the efficacy of TCM formula and single herb has always been the centre of discussion among scholars. Jin Xiaoling *et al.* found that the protective effect of each single herbs was weaker than that of the whole prescriptions when studying the protective effect of Sheng-Xian decoction on cardiomyocytes (Chen L et al., 2021). Compared with single herbs, Yi-Qi-Jie-Du-Hua-Yu formula plays a more important role in inhibiting the expression of Smad3 mRNA in glomerular mesangial cells and promoting the expression of Smad7 mRNA in mesangial cells (Wang et al., 2019). Similarly, Song Dongmei *et al.* confirmed that Tong-Xin-Luo formula has a better curative effect on vascular endothelial damage, collateral stasis and collateral dysfunction compared with single herbs (Song et al., 2013). The above studies have shown that the effect of TCM formula is better than that of single herbs. Single herbs are mostly used as prescriptions, and their role is to influence each other’s efficacy on the basis of the efficacy of single herbs, rather than just generating a simple additive effect of the single herbs (Zhou et al., 2017). So, why does the prescription play a more important role than the single herbs? Some scholars believe that the physical and chemical properties of single herbs are changed after compatibility, and that the efficacy will be enhanced to a certain extent (Zhang et al., 2020). Research has verified that the dissolution of the main ingredients in ZZHPD is affected by compatibility, and the anti-anxiety effect of ZZHPD is exerted based on the multi-component coordination (Chu et al., 2017). This effect is the biological performance of the TCM formula after intervention by active components under pathological conditions. According to the hypothesis of TCM, the multiple active phytochemical components in TCM formula can exert their medicinal effects through multiple molecules and multiple pathways, and may achieve better effects than a single herb. Therefore, it is of great significance to study the effective ingredients of TCM formula and explain the mechanism of compatibility (Wang et al., 2013). In the previous research, our research team found that when two kinds of TCM were decocted together, and apply the metabonomic data processing method to analysis the global compounds, and a variety of new formed unknown compounds were detected in co-decoction. Furthermore, a series of formulae of TCM were explored by our research team. Meanwhile, a novel concept of co-decoction reaction of TCM was put forward for the first time (Liu R et al., 2021; Wu et al., 2021; Zhang et al., 2021). In order to further explore the co-decoction phenomenon of the complex TCM formula consists of three or more kinds of herbal medicine, we carried out an exploratory study of Zhi-Zi-Hou-Po Decoction.

**FIGURE 1:**
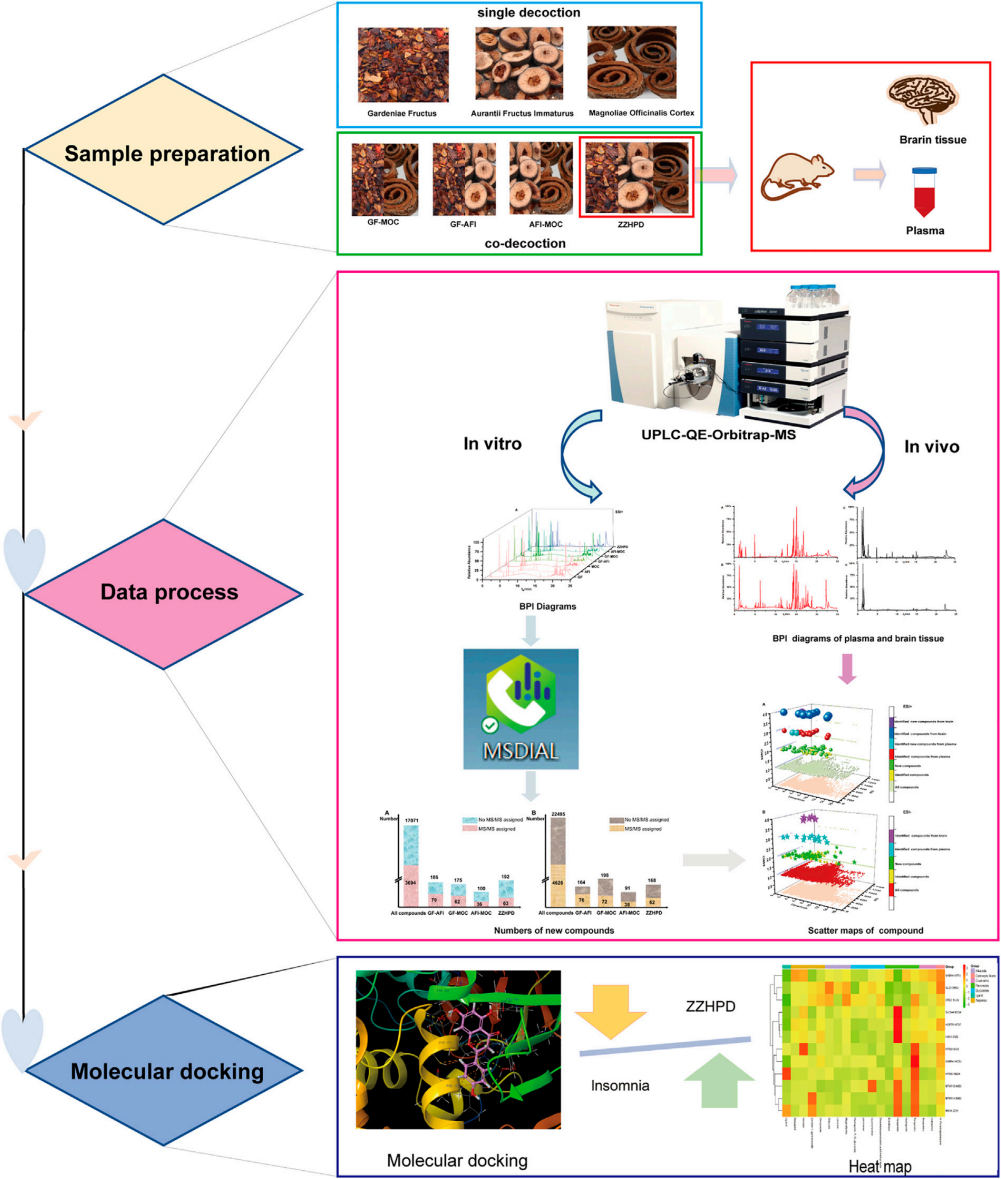
The research flowchart of the article.

TCM has always been an indispensable source of medicine for treating various diseases. However, due to the inherent chemical diversity and complexity of TCM, its’ safety and effectiveness in clinical application are limited. In the past 10 years, LC-MS has always been key tools for medicine research. The combination of LC and MS has made great contributions to the qualitative analysis of TCMs (Yu et al., 2021). The application of LC-MS in TCM mainly focuses on ingredients, metabolites and biologically active ingredients (Chen YH et al., 2021). This study has analysed the chemical components of a single decoction and co-decoction of ZZHPD based on UPLC-QE-Orbitrap-MS technology and MSDIAL software. We have also explored whether there are new compounds in the process of co-decoction, which may provide a unique idea for others. The components of ZZHPD absorbed into the blood and brain tissue were also analysed, to study whether the new compounds entered the body of mice and played a role in treating insomnia and depression. Finally, the binding ability of components absorbed into brain and insomnia-related targets was evaluated by molecular docking, revealing the mechanism of ZZHPD in treating insomnia and depression. The research flowchart is shown in Figure 1.

## Materials and Methods

### Chemicals and Reagents

*Gardeniae Fructus* (GF)*, Aurantii Fructus Immaturus* (AFI) *and Magnoliae Officinalis Cortex* (MOC) [Beijing Deshoutang Pharmaceutical Co., Ltd. (Beijing, China)], Acetonitrile, formic acid, methanol (LC-MS grade) (Thermo Fisher Scientific (China) Co. Ltd.), Watsons Distilled Water (Beijing, China).

### Preparation of Zhi-Zi-Hou-Po Decoction Extraction

The preparation method of all sample according to the original composition and preparation method of ZZHPD recorded in “*Shang Han Lun*”, with a little modification. The prescription includes GF 9 g, AFI 10 g, and MOC 62.4 g. Seven sample were prepared as follow: the single herb decoctions included three groups (GF/AFI/MOC), and the co-decoction included three groups of two-herb co-decoction (GF-AFI/GF-MOC/AFI-MOC) and a three-herb co-decoction (ZZHPD). The extraction preparation process of the seven sample was operated in parallel in order to reduce the difference as much as possible. Herbs of the seven sample were separately immersed in water (1:10, w/v) for 30 min, and then decocted at 95 ± 5°C for 1 h. Subsequently, the extraction was filtered, and the residue was decocted twice more. The ratios of the total weight of the herbs to the volume of water for the second and third decoction were 1:8 and 1:5, respectively. The three filtrates were combined and diluted to 250 ml to obtain the samples. The samples were centrifuged for 15 min at 12,000 r/min, and 5 µl of supernatant was taken for mass spectrometry analysis. Moreover, the three-herb co-decoction was concentrated to 45 ml by rotary evaporator dried in vacuum conditions, and stored at low temperature (4°C) for animal experiment.

### UPLC-QE-Orbitrap-MS

Chromatographic Separation Conditions: Waters HSS T3 UPLC C18 column (1.7 µm, 2.1 × 150 mm, Milford, MA, United States ); Mobile phase: 0.1% formic acid aqueous solution (A) and acetonitrile (B); Elution gradient: 0–2 min: 5% B; 2–17 min: 5–98% B; 17–20 min: 98–98% B; 20–23 min: 98–5% B; 23–25 min: 5% B; Column temperature: 40°C; Injection volume: 5 μl; Flow rate: 0.3 ml/min.

Mass Separation Conditions: Electrospray ionization source (ESI); Positive ions mode (20 V, 40 V, 60 V) and negative ions mode (30 V, 50 V, 70 V); Spray voltage 3800 V (**−**), 3200 V (**+**); Sheath gas: 35 arb; Auxiliary gas: 15 arb; Scan mode: the full scan/data-dependent two-stage scan (full scan/ddMS2); Scanning mass range: *m/z* 100–1,300 Da; Capillary temperature: 350°C.

### Animals

12 specific pathogen-free male ICR mice (4 weeks old, 25–30 g) were purchased from the Si pei fu (Beijing, China) Biotechnology Co., Ltd. (license number: SCXK (Jing) 2019-0010). Mice can freely obtain commercial standard chow diet and purified water, and are kept in an environmentally controlled room with a temperature of 25°C, a relative humidity of 60 ± 5%, and a 12 h light/dark cycle. Allow them to acclimate to the environment 7 days before the experiment. Animal experiments were approved by the Animal Care and Use Committee of Beijing University of Chinese Medicine (BUCM-4-20211111004-4067), and in accordance with the “Guidelines for the Care and Use of Laboratory Animals” published by the National Institutes of Health (NIH Publication No. 85-23, revised in 1996). All the mice were randomly divided into two groups (*n* = 6): a control group and a ZZHPD group. Mice of ZZHPD group were given a ZZHPD condensed liquid (1.81 g/ml) at the dose of three times of clinical dosage for 5 days.

### Preparation of Biological Sample

One hour after the last administration, blood samples were taken from the eyes of the mice, placed in an anticoagulation tube and stored in a refrigerator at 4°C. After blood collection, the mice were killed by cervical dislocation. The thoracic cavity was exposed, and the remaining blood in the mice was flushed with cardiac perfusion. Take out the brain tissue and homogenize with 1 ml of ultrapure water. Then the plasma and brain tissue were centrifuged at 4°C and 4,000 rpm for 15 min, and the supernatant was taken for subsequent operations. Three times the plasma volume of acetonitrile was added to the plasma supernatant to precipitate proteins. After acetonitrile was added, the mixture was vortexed for 3 min, and then the mixture was centrifuged at 8,000 rpm for 15 min at 4°C. The separated supernatant was dried with nitrogen, reconstituted with 100 μl of 50% methanol and vortexed for 3 min. The re-dissolved sample was centrifuged at 12,000 rpm for 15 min at 4°C, and the supernatant was taken as the final plasma sample. Perform the same operation on the brain tissue to obtain a brain tissue sample.

### Molecular Docking

Molecular docking needs to prepare the 3D structure of the compound and protein for molecular docking. The 3D structure of the brain component is obtained from PubMed (https://pubchem.ncbi.nlm.nih.gov/), and the 3D structure of the protein is obtained from the PDB (Protein Data Bank) database. The Maestro 11.8 software includes roughly four parts, processing ligands, optimizing protein structure, constructing binding pockets, and performing molecular docking. Firstly, The LigPrep panel needs to be used to process small molecules to obtain suitable ligands. Then in order to make these structures suitable for molecular docking, we use the protein preparation tool ProteinPreparation Wizard to pre-process the protein. The protein preparation process mainly includes three main steps: processing, modification and refinement. Fill missing side chains and/or loops, optimize hydrogen bond networks, change the protonation state of residues and ligands, repair possible conflicts that may occur when hydrogen is added or fill missing side chains, and minimize capabilities. Furthermore, the mating pocket is defined according to the binding site of the original ligand in the protein, which means that the centre and size of the original ligand will be used as the centre and size of the interface pocket by the receptor grid generation. Finally, the prepared ligand is docked with the optimized protein in a standard precision mode, so that the result of the docking can refer to the result of the original ligand.

### Data Processing and Analysis

The data collected by high-resolution mass spectrometry was processed using Compound Discoverer 3.1.1.12 software and MSDIAL software. Use Compound Discoverer software to perform peak alignment, peak detection, background subtraction and other operations to identify compounds. Due to the blindness of Compound Discoverer matching, it is necessary to combine M/Zcloud, Chemspider, M/Zvalue with literatures to confirm related compounds and ensure the accuracy of results. Furthermore, excimer ion peaks and unique fragment ions in the spectrogram have been used to deduce related structure of compounds, which were not matched in the database and have high response peaks in the spectrogram. Next, use Analysis Base File Converter software to convert the mass spectrum into a format that MSDIAL can recognize, normalize the data, and compare different compounds in the co-decoction process. The Jvenn website (http://jvenn.toulouse.inra.fr/app/example.html) and origin 2021 software were used to analyse and visualize the processed data, and Maestro 11.8 software was used to accomplish molecular docking.

## Results

### Compound Difference Between Single Decoction and Co-decoction

**FIGURE 2:**
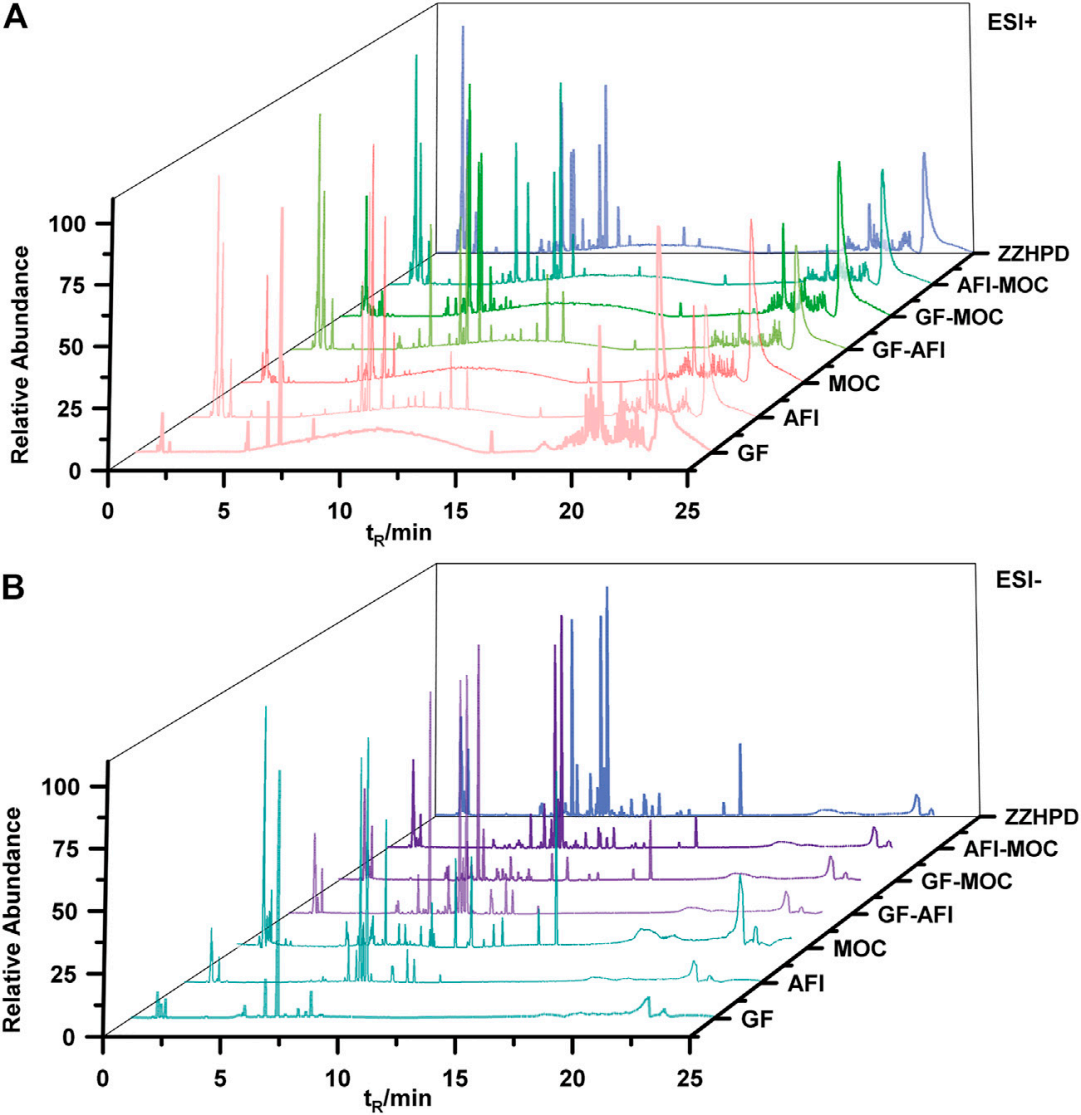
Typical BPI of GF, AFI, MOC and co-decoction under the positive ion mode (**A**) and negative ion mode (**B**).

Although qualitative and quantitative analysis is usually performed on certain components by UPLC-QE-Orbitrap-MS, information about new compounds is rarely considered by most scholars from the perspective of single decoction and co-decoction. We used UPLC-QE-Orbitrap-MS to determine the active components in the single-decoctions and co-decoctions, and drew a base peak intensity chromatogram (BPI) of seven samples (Figure 2). Meanwhile, by comparing standard compound databases and the literature, 106 main compounds in ZZHPD were predicted or identified, including phenolic acids, flavonoids and alkaloids (Additional file 1: Supplementary Table S1).

**FIGURE 3:**
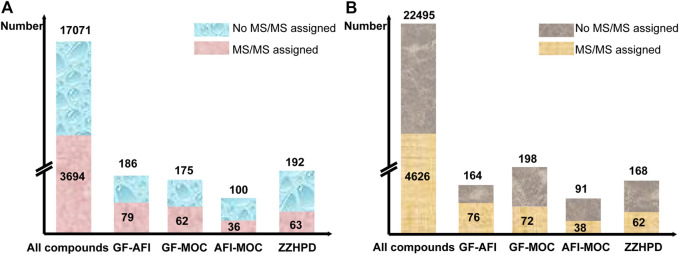
Data processing results of MSdial software in the positive mode(**A**) and in the negative mode(**B**).

In this research, the MSDIAL software was used to normalize all the mass spectra data collected in the single decoctions and co-decoctions samples, and compounds were labelled with retention-time and accurate mass. Subsequently, eliminate peaks without secondary fragments in the results, and construct an extracted ion chromatogram in order to further reduce false-positive results. After analysing and processing the mass spectrum data of the ZZHPD co-decoction, it was found that a total of 17,071 peaks were detected in the positive ion mode, and a total of 22,495 peaks were detected in the negative ion mode. Then cross-analyse the data of seven samples in the positive ion mode, compare single decoction and co-decoction, and screen new compounds with a normalized value greater than 10^−5^. The data in the negative ion mode were processed in the same way as in the positive ion mode process. We will be shocked by the findings that there are 653 compounds from the co-decoction including two-herb and three-herb in the positive mode, and these compounds do not exist in the single decoction. Only 240 compounds showed Ms/Ms assigned after normalization by MSDIAL, including 79 compounds from the co-decoction of GF-AFI, 62 compounds from GF-MOC, 36 compounds from AFI-MOC, and 63 compounds from ZZHPD, which indicates that new compounds appear through certain chemical reactions during co-decoction (Figure 3A.). Similarly, there were 621 compounds from the co-decoction including two-herb and three-herb in the negative mode, with only 248 compounds showing Ms/Ms assigned after normalization, including 76 compounds from GF-AFI, 72 compounds from GF-MOC, 38 compounds from AFI-MOC, and 62 compounds from ZZHPD (Figure 3B.).

**FIGURE 4:**
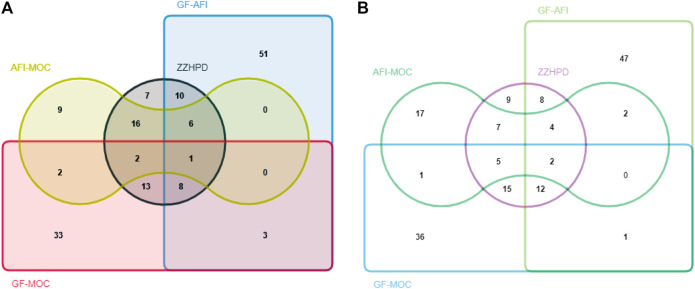
Traceability of new compounds under the positive ion mode (**A**) and negative ion mode (**B**).

125 new compounds have appeared in ZZHPD, and their mass spectra are shown in Additional file 2 Supplementary Table S2. The source of the new compounds produced by the three herbs co-decoction was then traced. Seven compounds are produced by the interaction of three herbs and the remaining 56 new compounds were derived from the two herbs co-decoctions (10 new compounds belonging to ZZHPD/GF-AFI, 13 new compounds belonging to ZZHPD/GF-MOC, 16 new compounds belonging to ZZHPD/AFI-MOC, eight new compounds belonging to ZZHPD/GF-AFI/GF-MOC, six new compounds belonging to ZZHPD/GF-AFI/AFI-MOC, 2 new compounds belonging to ZZHPD/GF-MOC/AFI-MOC, and 1 new compound belonging to ZZHPD/GF-AFI/GF-MOC/AFI-MOC) in the positive ion mode. It was found that nine new compounds were only derived from the three herb co-decoction, and the remaining 53 new compounds were derived from the two herb co-decoctions (8 new compounds belonging to ZZHPD/GF-AFI, 15 new compounds belonging to ZZHPD/GF-MOC, seven new compounds belonging to ZZHPD/AFI-MOC, 12 new compounds belonging to ZZHPD/GF-AFI/GF-MOC, four new compounds belonging to ZZHPD/GF-AFI/AFI-MOC, five new compounds belonging to ZZHPD/GF-MOC/AFI-MOC, and 2 new compounds belonging to ZZHPD/GF-AFI/GF-MOC/AFI-MOC) in the negative ion mode. The results are shown in Figure 4. Unfortunately, Due to the incomplete structure identification information, there is insufficient evidence to accurately identify new compounds.

### Analysis of the Components of ZZHPD Absorbed Into the Blood and Brain, Based on UPLC-QE-Orbitrap-MS

**FIGURE 5:**
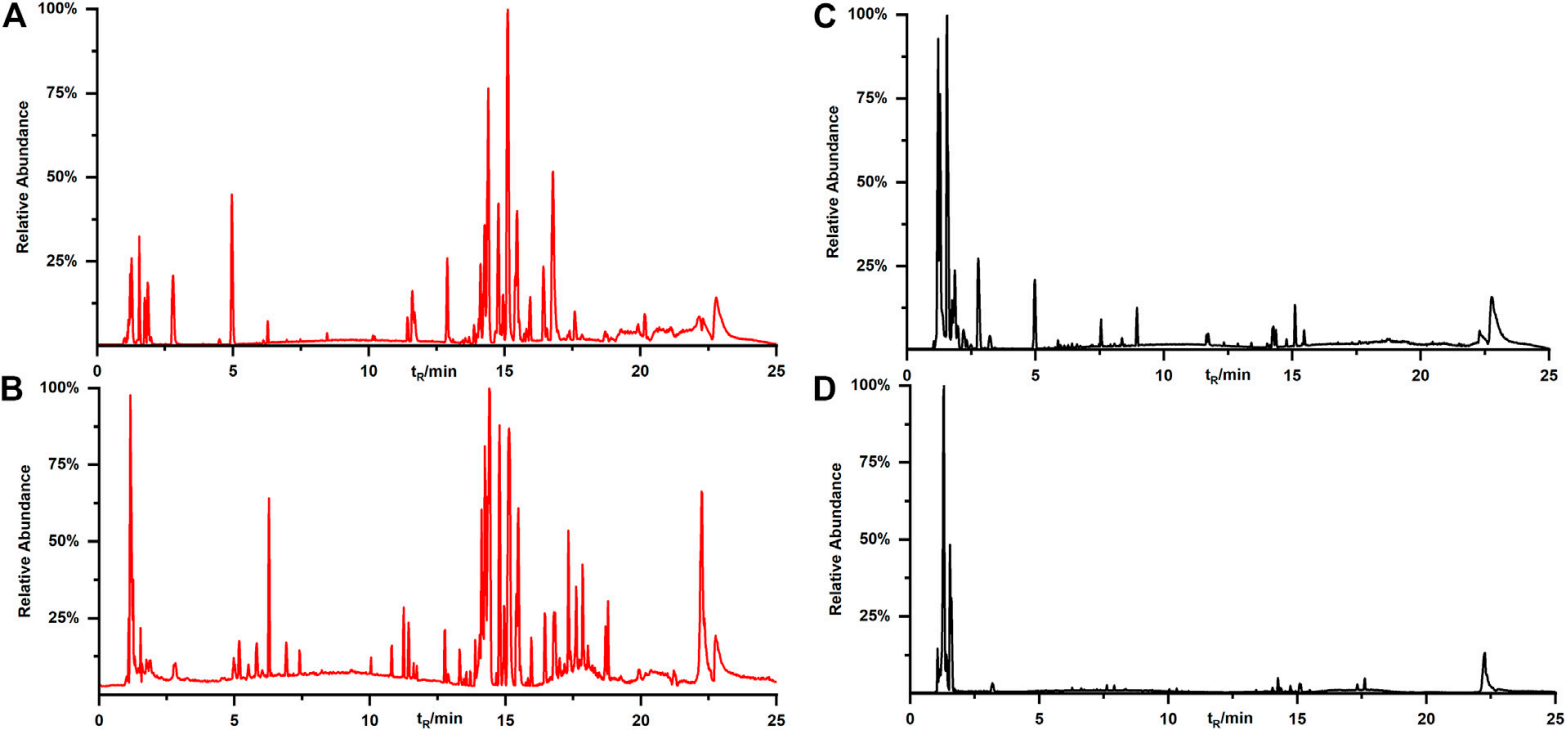
The BPI of blood components of ZZHPD under the positive ion mode (**A**) and negative ion mode (**B**); The BPI of brain components of ZZHPD under the positive ion mode (**C**) and negative ion mode (**D**).

By comparing literatures, spectres, and results from the MSDIAL, we have initially identified the components of ZZHPD *in vitro*, and then verified whether the components *in vitro* were enriched in the blood and brain tissue, and played a pharmacological effect. The BPI diagram of drug-containing plasma and the brain extract samples under the negative ion mode and positive ion mode were shown in Figure 5.

**FIGURE 6:**
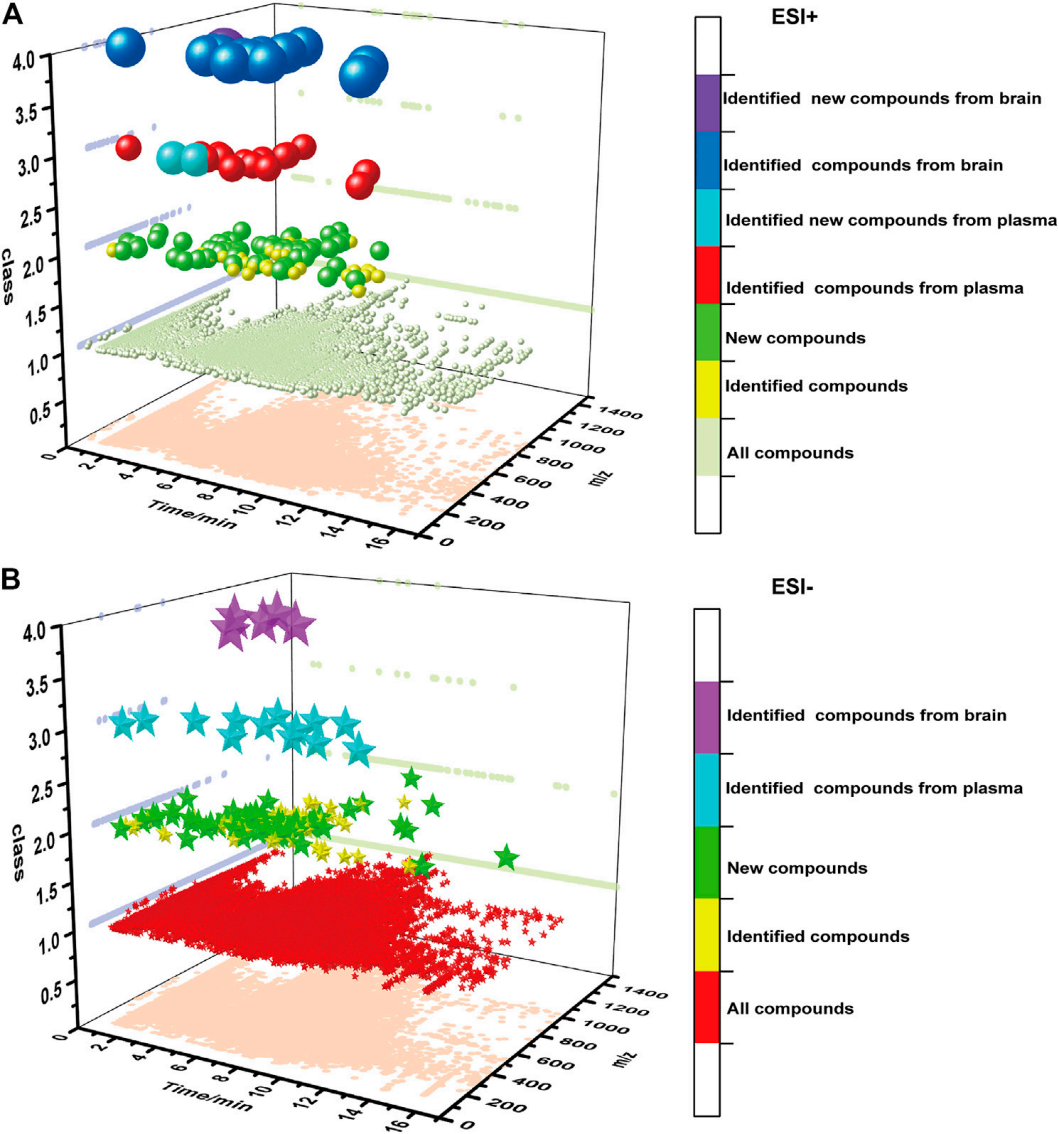
Information of the chemical compounds of Zhi-Zi-Hou-Po decoction under the positive ion mode(**A**) and negative ion mode (**B**).

In total, 30 compounds of ZZHPD were screened in plasma, including 28 known compounds and two new compounds under the positive and negative ion mode. And, 18 known compounds and one new compound were absorbed into brain tissue under the positive and negative ion mode. Origin software was used to draw the chromatographic scatter diagrams of all components of ZZHPD, identified components of ZZHPD, new components, blood components and brain components, as shown in Figure 6. More information was shown in additional files.

The components entering the blood and the brain tissue were far fewer than those outside the body. There are two reasons: the existence of the blood-brain barrier (BBB) and the influence of the intestinal flora. Specifically, the BBB, one of the internal barriers in the body regulating innate immunity, restrict drug delivery during the treatment of central nervous system diseases. TCM contains complex chemical components, and only a few chemical components of TCM, such as polar or lipid soluble small molecules, can exert therapeutic effects on central nervous system diseases through BBB (Jin et al., 2013). Furthermore oral administration of TCM is inevitably interacted with intestinal flora (Yi et al., 2021), and the interaction between the active ingredients of TCM and intestinal flora has become research hotspots in recent years. Some biologically active ingredients can’t pass through the intestine, resulting in low bioavailability. The components of TCM are metabolized or biotransformed by the intestinal microbiota, thereby promoting the production and absorption of new active compounds. Meanwhile, the function of diseased organs/tissues is regulated by the ingredients of TCM by influencing the composition and structure of intestinal flora (Gong et al., 2020). Although there are few new compounds found in brain and blood, they still have certain research value and significance.

### Results of Molecular Docking Experiment

**FIGURE 7:**
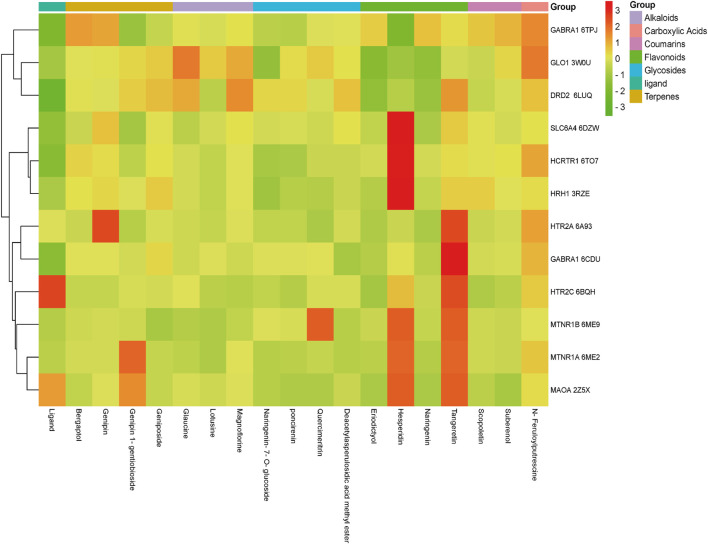
The heat map of molecular docking results.

According to the key proteins of some cutting-edge drugs in fighting insomnia and depression, 12 proteins including GABA receptor, serotonin, histamine, dopamine and orexins were screened out. Among them, the Dong Ying-Jie team found that Modified Suan-Zao-Ren Decoction can effectively increase the content of 5-HTR1A protein in the hypothalamus of insomnia mice and down-regulate the expression of OX2R, indicating that Modified Suan-Zao-Ren Decoction can effectively improve insomnia (Dong et al., 2021). Combined with the RCSB protein database (PDB), the proteins were finally obtained according to the source and resolution of proteins and the influence of the original ligand on the protein. After analysis and comparison, 12 proteins were obtained as targets related to insomnia and depression (Table 2.). All the components absorbed by the brain are used for docking with proteins. In order to verify the accuracy of the results, it is necessary to evaluate the original ligands in the protein crystal structure with active compounds and draw the results into a heat map (Figure 7.).

**TABLE 2:** Molecular docking results of 18 prototype components absorbed into the brain from Zhi-Zi-Hou-Po decoction.

Compound of brain	Classfication	HCRTR1 6TO7	GABRA1 6TPJ	MTNR1B 6ME9	MTNR1A 6ME2	GLO1 3W0U	DRD2 6LUQ	HTR2A 6A93	HTR2C 6BQH	SLC6A4 6DZW	GABRA1 6CDU	MAOA 2Z5X	HRH1 3RZE
Ligand	Ligand	−9.33	−8.96	−9.54	−8.62	−10.28	−9.63	−4.73	0.24	−8.76	−7.85	−1.92	−8.28
Bergaptol	Terpenes	−5.55	−5.43	−7.99	−7.43	−8.23	−5.87	−5.20	−4.47	−6.77	−5.38	−5.46	−6.28
Genipin	Terpenes	−5.77	−5.56	−7.78	−7.42	-8.19	−6.04	−	−4.44	−5.00	−5.32	−4.58	−6.04
Genipin 1-gentiobioside	Terpenes	−7.26	−8.13	−7.95	—	−7.76	−5.35	−5.91	−4.00	−7.99	−5.67	−1.49	−6.79
Geniposide	Terpenes	−5.88	−7.20	−10.19	−8.08	−7.38	−4.87	−4.91	−4.20	−5.99	−4.84	−5.26	−5.65
Deacetylasperulosidic acid methyl ester	Glycosides	−6.81	−6.70	−9.27	−8.99	−8.09	−5.03	−4.99	−4.02	−5.94	−6.89	−5.23	−7.27
Naringenin-7-O-glucoside	Glycosides	−8.05	−7.41	−7.31	−8.92	−11.00	−5.48	−5.53	−4.57	−6.53	−5.46	−5.89	−8.92
Poncirenin	Glycosides	−7.91	−7.46	−7.50	−8.79	−7.96	−5.52	−5.50	−5.09	−6.37	−5.42	−6.16	−7.94
Quercimeritrin	Glycosides	−6.78	−6.82	—	−8.14	−7.41	−6.10	−6.32	−4.02	−6.69	−5.27	−6.29	−8.07
Eriodictyol	Flavonoids	−6.60	−6.24	−8.16	−8.73	−11.64	−8.04	−6.14	−5.45	−6.97	−6.56	−6.32	−8.09
Hesperidin	Flavonoids	-	−8.94	—	—	−10.50	−6.97	−5.32	−2.69	—	−5.21	—	—
Naringenin	Flavonoids	−6.55	−6.07	−8.33	−9.21	−11.06	−7.77	-6.30	−4.38	−7.75	−6.31	−6.42	−7.43
Pentamethoxyflavone	Flavonoids	−5.81	−6.47	—	—	−8.69	−4.09	—	—	−5.21	—	—	−5.81
Scopoletin	Coumarins	−6.07	−6.14	−7.99	−7.55	−8.98	−6.54	−5.22	−5.16	−5.98	−5.67	−5.84	−5.86
Suberenol	Coumarins	−5.93	−5.80	−8.19	−7.77	−7.72	−6.14	−4.94	−4.79	−6.47	−5.56	−6.50	−6.60
N-Feruloylputrescine	Carboxylic Acids	−4.14	−5.12	−6.97	−4.90	−5.11	−4.97	−2.51	−3.01	−5.93	−3.97	−3.96	−6.26
Glaucine	Alkaloids	−6.62	−6.67	−9.65	−8.47	−5.05	-4.50	−5.08	−3.80	−7.27	−5.73	−4.69	−7.06
Lotusine	Alkaloids	−7.06	−6.91	−9.74	−9.36	−7.37	-6.87	−5.34	−4.88	−6.51	−5.41	−4.99	−7.58
Magnoflorine	Alkaloids	−6.19	−6.58	−8.54	−6.73	−6.42	-3.91	−4.79	−4.95	−5.89	−5.75	−4.60	−6.61

**FIGURE 8:**
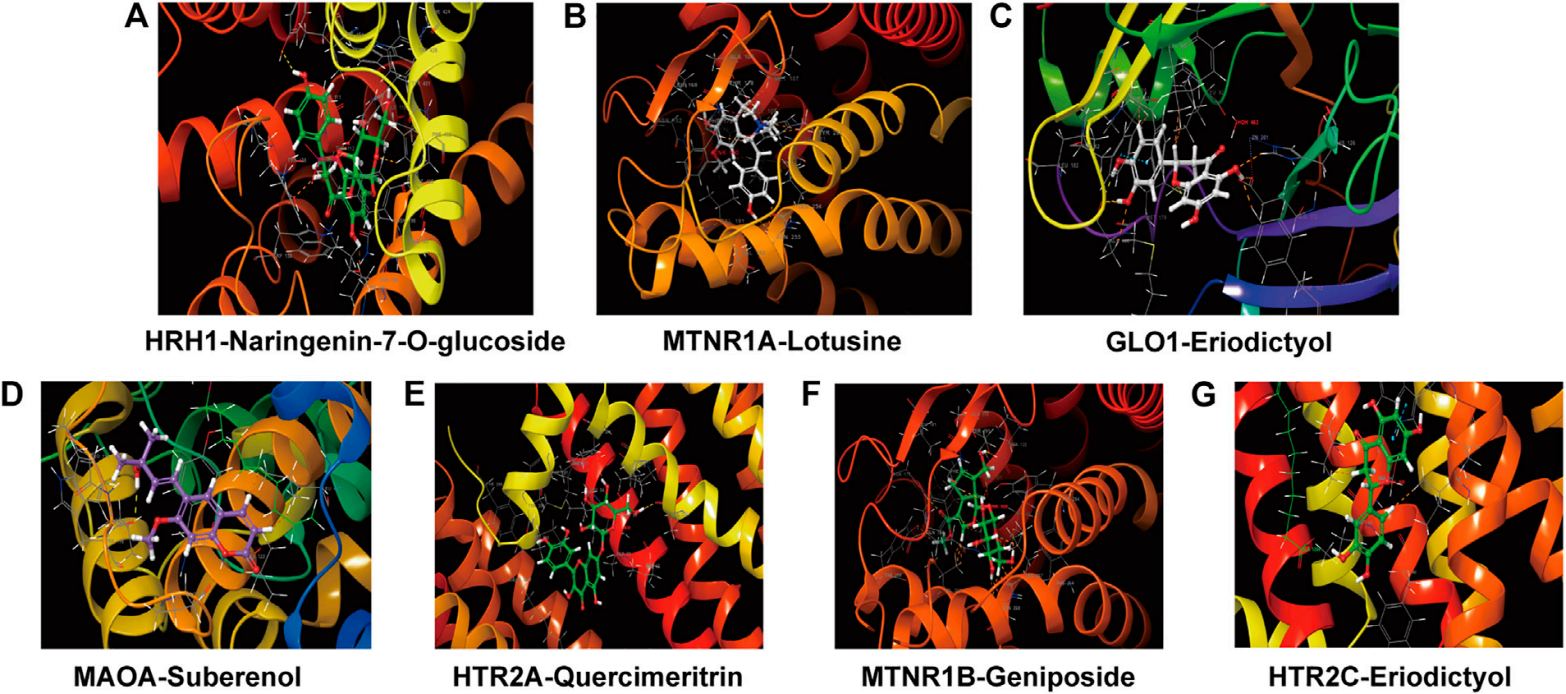
Molecular docking of representative compounds and related receptors. The 3D diagram of the docking of HRH1 with Naringenin-7-O-glucoside **(A)**, MTNR1A with Lotusine **(B)**, GLO1 with Eriodictyol **(C)**, MAOA with Suberenol **(D)**, HTR2A with Quercimeritrin **(E)**, MTNR1B with Geniposide **(F)**, HTR2C with Eriodictyol **(G)**.

According to the results of molecular docking, there are seven proteins have higher binding affinity to the brain components than the original ligand, such as eriodictyol with HCRTR1, MTNR1A, GABRA1, HCRTR1, MAOA, GLO1 and HTR2A; naringenin-7-O-glucoside with HCRTR1, MTNR1A, MAOA, GLO1, HTR2A and HRH1; naringenin with MAOA, HCRTR1, MTNR1A, GABRA1, HCRTR1, GLO1 and HTR2A; as well as lotusine with MTNR1B, HCRTR1, MTNR1A, GABRA1, MAOA and HTR2A. The result shows that ZZHPD may play an important role in treating insomnia. The docking results of components absorbed into brain and related receptors are shown in Figure 8.

## Discussion

With the improvement of modern science and technology, humans also need continuous progress. Particularly as pharmaceutical analysts, we should make full use of advanced equipment and different methods to settle various problems. People have been discovering new things and expanding their ideas since ancient times, and the emergence of something new often drives the development of things. In this study, whether the new compounds exist in the co-decoction of ZZHPD is analysed based on high-resolution mass spectrometry and MSDIAL software. The MSDIAL software was launched as a universal program for untargeted metabolomics, which has many features, including data-independent MS/MS and spectral deconvolution for both GC/MS, conversion of raw data to analytical data and streamlined criteria for peak identification. The software-assisted data processing method had significantly improved the efficiency of MS data interpretation for complex TCM systems. Overall, 125 unique compounds were found in the co-decoction via spectral comparison. Mass spectrometry information (such as retention time, molecular weight and fragments) facilitated in the identification of the new compounds in plasma and brain tissue.

Insomnia and depression are a very common disease. The continuous medicinal needs of patients with insomnia have focused a lot of attention on the discovery of insomnia drugs (Feizi et al., 2019). Melatonin (melatonin receptor agonist), Benzodiazepine receptor agonists (BZRAs) and histamine antagonists, and orexin receptor antagonists are the most popular drugs in treating insomnia (Dujardin et al., 2020). BZRAs, including benzodiazepine related drugs (BZRDs) and benzodiazepines (BZDs), act on the gamma-aminobutyric acid (GABA) receptor and are the mainstay treatments for insomnia (Huang et al., 2021). Ramelteon, as a sleep-promoting agent, can reduce the arousal promotion signal from the suprachiasmatic nucleus (mainly through the melatonin MT1 receptor), and affect sleep time through the melatonin MT2 receptor. The ability of mirtazapine to antagonize 5HT2C, 5-HTA and 5-HT3 receptors causes the remaining serotonin concentration to interact with free 5-HT1 receptors. The interaction with 5-HT1 receptors (especially 5-HT1A receptors) have antidepressant effects. It also exhibits moderate or weak antagonism to peripheral α1-adrenergic receptors and muscarinic receptors (Jeong and Bahk, 2014). Reserpine, a vesicle reuptake inhibitor, can deplete neurotransmitters such as norepinephrine and serotonin, and cause physiological changes (Becker et al., 2021). Amitriptyline, as a tricyclic antidepressant, has been approved by the FDA in treating adults with depression (Di Pierro and Settembre, 2015; Anderson et al., 2017). Amitriptyline is also used off-label to treat anxiety, chronic pain syndrome and insomnia. Based on the important role of orexin in sleep/wake regulation, orexin receptor antagonists have become the focus of new therapies for the treatment of insomnia (Patel et al., 2015; Ito et al., 2021). Although the western medicine plays an important role in the treatment of insomnia, its side effects such as drug resistance and addiction cannot be ignored at present. (Wang et al., 2020).

TCM has a long history of treating insomnia and depression with fewer side effects. Many Chinese herbs and prescriptions have good effects on treating anxiety and insomnia (Zhao, 2019). The TCM prescription library for the treatment of insomnia had been built using an auxiliary platform of the inheritance of TCM. Scientists found that the most frequently used medicine was *Ziziphi Spinosae Semen*(*Ziziphus jujuba var. spinosa (Bunge) Hu ex H.F.Chow)*, followed by *Poria*(*Poria cocos*(*Schw.)Wolf)*, *Glycyrrhizae Radix Et Rhizoma* (*Glycyrrhiza* uralensis Fisch)and *Polygoni Multiflori Caulis(Polygonum multiflorum Thunb.) (* Ma et al., 2021). As a classic Chinese prescription, ZZHPD has been proven to have a good effect on depression-like symptoms with few side effects (Sun et al., 2021). However, due to the complex components and multi-target nature of ZZHPD, the mechanism in treating insomnia is still unclear. Studies have shown that the mechanism of ZZHPD in treating insomnia is mainly related to GABA synthase and GABA metabolizing enzymes (Feizi et al., 2019). Naringenin can effectively improve the harm caused by an efavirenz-induced sleep-like disorder in the midbrain of white albino mice (Olufunke et al., 2020). An infusion of flowers of several species of the Citrus genera is used as a sedative to treat insomnia, and hesperidin may participate in the function of adenosine receptors and exert a sedative effect (Guzmán-Gutiérrez and Navarrete, 2009; Adhikari-Devkota et al., 2019). Geniposide ameliorates the depression-like behaviour induced by chronic unpredictable mild stress, through inhibition of ceramide-PP2A signalling via the PI3K/Akt/GSK3β axis (Wang et al., 2021). Furthermore, LC-MS technology was used to identify components absorbed into the brain, and 18 compounds were found, which indicated that these prototype components play a key role in anti-insomnia activities. There are four terpenes, 4 glycosides, 4 flavonoids, 3 alkaloids, 2 coumarins and 1 organic acid, specifically including bergaptol, deacetylasperulosidic acid methyl ester, eriodictyol, genipin, genipin 1-gentiobioside, geniposide, glaucine, hesperidin, lotusine, magnoflorine, naringenin, naringenin-7-O-glucoside, n-Feruloylputrescine, poncirenin, quercimeritrin, scopoletin and suberenol. However, there are only four main ways for pharmaceutical ingredients to penetrate the BBB: 1) small water-soluble molecules directly diffuse through the intercellular space; 2) transmembrane diffusion of fat-soluble molecules; 3) pinocytosis mediated by specific receptors; 4) activation of specific carrier channels and enzyme systems (Jin et al., 2013). TCM contains complex components, and the main substances that can penetrate the BBB are currently polar or fat-soluble small molecules. It is worth noting that, as a macromolecular flavonoid compound, hesperidin can penetrate the BBB, but the penetration mechanism needs further research. Besides, the results of molecular docking indicated that GABRA1 has a strong binding power with naringenin and hesperidin, and HCRTR1 has strong binding power with naringenin-7-O-glucoside, poncirenin, and genipin 1-gentiobioside. The research provides a reference for research in treating insomnia and depression with ZZHPD *in vivo* and *in vitro.*

## Conclusion

In this study, an LC-MS method was developed to detect the ZZHPD components, and 125 new compounds were investigated by analysing the MS information of the co-decocting of ZZHPD for the first time. Although there are few new compounds absorbed into the plasma and brain tissue, their effects could not be denied. Molecular docking also showed that Lotusine, Eriodictyol, Naringenin and Naringenin-7-O-glucoside are active ingredients acting on melatonin receptors, GABA receptors and serotonin-related pathways. The research takes the material changes of the ZZHPD complex reaction system as the breakthrough point to provide new ideas for the development of TCM, which is based on the theory of Chinese medicine co-decoction.

## Data Availability

The original contributions presented in the study are included in the article/Supplementary Material, further inquiries can be directed to the corresponding authors.

## Abbreviations

ZZHPD, Zhi-Zi-Hou-Po Decoction; LC-MS, liquid chromatography-mass spectrometry; GF, Gardeniae Fructus; AFI, Aurantii Fructus Immaturus; MOC, Magnoliae Officinalis Cortex; TCM, traditional Chinese medicine; BBB, blood-brain barrier; BZRAs, Benzodiazepine receptor agonists; BZDs, benzodiazepines BZRDs, benzodiazepine related drugs; GABA, act on the gamma-aminobutyric acid.
