# Supplementary material for: Discovery of the Potential Novel Pharmacodynamic Substances From Zhi-Zi-Hou-Po Decoction Based on the Concept of Co-Decoction Reaction and Analysis Strategy
Source: Front Pharmacol. 2022 Jan 13;12:830558. doi: 10.3389/fphar.2021.830558 (PMC8793358; doi:10.3389/fphar.2021.830558)
Supplement: Supplementary file 1 [file Table1.DOCX]

**Table S1.** The identified Zhi-Zi-Hou-Po decoction effective extraction constituents by UPLC-QE-Orbitrap-MS.

| **t_R_/min** | **Theoretical m/z** | **Measured *m/z*** | **Error (ppm)** | **Adduct** | **MS/MS** | **Formula** | **Identification** | **Classification** | **Blood** | **Brain** |
| --- | --- | --- | --- | --- | --- | --- | --- | --- | --- | --- |
| 1.19 | 193.07048 | 193.07121 | 3.78 | [M+H]^+^ | 147.0651;139.0388; 129.0546; 111.0442; 95.0495; 69.0341 | C_7_H_12_O_6_ | Quinic acid | GF | No | No |
| 1.20 | 191.01917 | 191.01972 | 2.88 | [M-H]^−^ | 191.0556; 173.0444; 127.0390; 111.0439; 85.0283 | C_6_H_8_O_7_ | Citric acid | AFI | Yes | No |
| 1.37 | 168.10191 | 168.10191 | -0.03 | [M+H]^+^ | 150.0912;135.0677; 119.0493;91.0546 | C_9_H_13_NO_2_ | Synephrine | AFI | No | No |
| 1.50 | 268.10379 | 268.10403 | 0.90 | [M+H]+ | 268.1034; 136.0617 | C_10_H_13_N_5_O_4_ | Adenosine | GF/AFI/MOC | Yes | Yes |
| 1.51 | 287.07736 | 287.07720 | -0.56 | [M-H]^−^ | 149.0235; 125.0234 | C_12_H_16_O_8_ | 1-β-D-Glucopyranosyloxy-3,5- dihydroxybenzene or isomer | AFI | Yes | No |
| 1.55 | 209.08113 | 209.08084 | -1.41 | [M-H]^-^ | 191.0192; 85.0283 | C_11_H_14_O_4_ | Sinapyl alcohol | MOC | No | No |
| 1.82 | 161.02370 | 161.02441 | 4.41 | [M-H]^−^ | 161.0237; 133.0287; 117.0336 | C_9_H_6_O_3_ | 7-Hydroxy-2H-chromen-2-one | AFI | No | No |
| 3.06 | 391.12482 | 391.12458 | -0.61 | [M-H]^−^ | 229.0717;185.0812;167.0706 | C_16_H_24_O_11_ | Shanzhiside | GF | No | No |
| 3.28 | 167.03410 | 167.03498 | 5.28 | [M-H]^−^ | 152.0106; 108.0205 | C_8_H_8_O_4_ | 3-Methoxy-4-hydroxybenzoic acid | MOC | No | No |
| 3.34 | 373.11423 | 373.11402 | -0.56 | [M-H]^−^ | 211.0612; 149.0599;123.0441 | C_16_H_22_O_10_ | Geniposidic acid | GF | Yes | No |
| 3.52 | 153.01865 | 153.01933 | 4.44 | [M-H]^−^ | 153.0184; 109.0283 | C_7_H_6_O_4_ | Gentisic acid | GF | No | No |
| 4.55 | 353.08801 | 353.08781 | -0.58 | [M-H]^−^ | 353.0876;191.0555 | C_16_H_18_O_9_ | Chlorogenic acid | GF | No | No |
| 4.56 | 389.10950 | 389.10893 | -1.46 | [M-H]^−^ | 345.1190; 209.0447; 165.0550; 139.0391 | C_16_H_22_O_11_ | Deacetylasperulosidic acid | GF | No | No |
| 4.76 | 345.15570 | 345.15549 | -0.61 | [M-H]^−^ | 165.0912; 121.4539 | C_16_H_26_O_8_ | Jasminoside B/D | GF | No | No |
| 4.78 | 449.12964 | 449.13006 | 0.94 | [M+HCOO]^-^ | 241.0717; 191.0342; 161.0236; 127.0390; 101.0232 | C_17_H_24_O_11_ | Deacetylasperulosidic acid methyl ester | GF | Yes | Yes |
| 4.79 | 403.12479 | 403.12458 | -0.52 | [M-H]^−^ | 241.0722; 223.0612;139.0391; 127.0390 | C_17_H_24_O_11_ | Gardenoside | GF | No | No |
| 4.80 | 312.15964 | 312.15942 | -0.70 | [M] ^+^ | 312.1592; 299.7648; 269.1170; 237.0905; 209.0959 | C_19_H_22_NO_3_ | 3,4-dehydromagnocurarine | MOC | Yes | No |
| 4.85 | 369.15112 | 369.15198 | 2.33 | [M + Na] ^+^ | 369.1516; 207.0991; 143.1481 | C_16_H_26_O_8_ | Picrocrocinic acid | GF | No | No |
| 5.22 | 429.13739 | 429.13673 | -1.54 | [M + Na] ^+^ | 429.1357; 267.0842 | C_17_H_26_O_11_ | Shanzhiside methyl ester | GF | No | No |
| 5.48 | 342.17010 | 342.16998 | -0.35 | [M] ^+^ | 297.1119; 282.0882; 265.0856; 237.0907; | C_20_H_24_NO_4_ | Magnoflorine | MOC | Yes | Yes |
| 5.52 | 265.15466 | 265.15467 | 0.03 | [M+H]^+^ | 248.1281; 177.0546; 145.0284; 117.0338 | C_14_H_20_N_2_O_3_ | N-Feruloylputrescine | MOC | Yes | Yes |
| 5.54 | 639.19318 | 639.19305 | -0.20 | [M-H]^−^ | 529.1580; 459.1523; 367.1015; 313.0931; 161.0236; 179.0343 | C_29_H_36_O_16_ | Plantamajoside or isomer | MOC | No | No |
| 5.56 | 353.08795 | 353.08781 | -0.41 | [M-H]^−^ | 191.0555;179.0339;161.0236 | C_16_H_18_O_9_ | Neochlorogenic acid | GF | No | No |
| 5.57 | 355.10239 | 355.10235 | -0.11 | [M+H]^+^ | 163.0389; 145.0284; 135.0441; 117.0336;111.0421 | C_16_H_18_O_9_ | 3-Caffeoylquinic acid | GF | No | No |
| 5.75 | 595.18817 | 595.18742 | -1.26 | [M+HCOO]^-^ | 225.0769; 207.0659; 123.0441 | C_23_H_34_O_15_ | Genipin 1-gentiobioside | GF | Yes | Yes |
| 5.78 | 549.18243 | 549.18249 | 0.11 | [M-H]^−^ | 207.0658;123.0441;101.0232 | C_23_H_34_O_15_ | Genipin-1-β- D -gentiobioside | GF | No | No |
| 5.82 | 801.24670 | 801.24533 | -1.71 | [M+HCOO]^-^ | 621.2047; 477.1621; 383.0997; 179.0344; 161.0236 | C_34_H_44_O_19_ | Magnoloside I | MOC | No | No |
| 5.84 | 314.17474 | 314.17507 | 1.05 | [M] ^+^ | 269.1169; 237.0908;175.0752;107.0494 | C_19_H_24_NO_3_ | Lotusine/Magnocurarine/Oblongine | MOC | Yes | Yes |
| 5.93 | 343.10345 | 343.10345 | 0.00 | [M-H]^−^ | 197.0450; 182.0215; 153.0549; 138.0313; 123.0077 | C_15_H_20_O_9_ | Syringic acid 4-O-α-L-rhamnopyranoside | MOC | No | No |
| 5.95 | 477.16141 | 477.16136 | -0.10 | [M-H]^−^ | 183.0657; 168.0420; 89.0232 | C_20_H_30_O_13_ | Kelampayoside A or isomer | MOC | No | No |
| 5.97 | 785.25134 | 785.25096 | -0.48 | [M-H]^−^ | 623.2199; 477.1616; 315.1087; 221.0663; 179.0343; 161.0236 | C_35_H_46_O_20_ | Magnoloside F | MOC | No | No |
| 6.07 | 771.23621 | 771.23532 | -1.16 | [M-H]^−^ | 609.2044; 477.1617; 315.1083; 179.0554; 161.0236 | C_34_H_44_O_20_ | Magnoloside G | MOC | No | No |
| 6.08 | 593.15186 | 593.15119 | -1.12 | [M-H]^−^ | 473.1094; 383.0778; 353.0670 | C_27_H_30_O_15_ | Vicenin-2 | AFI | No | No |
| 6.24 | 785.25183 | 785.25096 | -1.11 | [M-H]^−^ | 623.2192;161.0236 | C_35_H_46_O_20_ | Magnoloside B | MOC | No | No |
| 6.26 | 433.13474 | 433.13460 | -0.32 | [M+HCOO]^-^ | 225.0765;123.0440;101.0232 | C_17_H_24_O_10_ | Geniposide | GF | Yes | Yes |
| 6.27 | 225.07623 | 225.07570 | -2.35 | [M-H]^−^ | 101.0233;123.0441;68.9970; 207.0657; 147.0442 | C_11_H_14_O_5_ | Genipin | GF | Yes | Yes |
| 6.32 | 623.16199 | 623.16176 | -0.37 | [M-H]^−^ | 503.1204; 413.0881; 383.0777; 312.0641; 161.0237 | C_28_H_32_O_16_ | Aigenin 6-C-β-D-glucosyl-8-C-β-Dglucoside or isomer | AFI | No | No |
| 6.49 | 623.19760 | 623.19814 | 0.87 | [M-H]^−^ | 461.1663; 161.0234; 315.1085; 133.0284 | C_29_H_36_O_15_ | Acteoside or isomer | MOC/GF | No | No |
| 6.51 | 623.19788 | 623.19814 | 0.42 | [M-H]^−^ | 461.1663;315.1085;161.0234 | C_29_H_36_O_15_ | Methyl hesperidin | AFI | No | No |
| 6.58 | 330.16998 | 330.16998 | 0.00 | [M+H]^+^ | 330.1697; 192.1018; 175.0754; 137.0597 | C_19_H_23_NO_4_ | Reticuline | MOC | No | No |
| 6.83 | 609.14691 | 609.14611 | -1.32 | [M-H]^−^ | 300.0276; 151.0029; 301.0354 | C_27_H_30_O_16_ | Rutin | GF | No | No |
| 7.05 | 463.08884 | 463.08819 | -1.40 | [M-H]^−^ | 300.0278; 151.0029 | C_21_H_20_O_12_ | Isoquercitrin | GF | No | No |
| 7.07 | 595.16632 | 595.16575 | -0.96 | [M+H]^+^ | 287.0546; 153.0180; 121.0287; 137.0229 | C_27_H_30_O_15_ | Nicotiflorin | GF | No | No |
| 7.08 | 771.23820 | 771.23532 | -3.74 | [M-H]^−^ | 301.0718; 286.0487 | C_34_H_44_O_20_ | Hesperetin-Glc-Glc-Rha or isomer | AFI | No | No |
| 7.09 | 595.16693 | 595.16684 | -0.15 | [M-H]^−^ | 433.1508; 373.1296; 151.0028 | C_27_H_32_O_15_ | Eriocitrin | AFI | No | No |
| 7.10 | 465.10303 | 465.10275 | -0.60 | [M+H]^+^ | 465.1727; 303.0497; 85.0289; | C_21_H_20_O_12_ | Isoquercitrin | GF | No | No |
| 7.11 | 593.15173 | 593.15119 | -0.91 | [M-H]^−^ | 447.0936;285.0406; 151.0025 | C_28_H_34_O_14_ | Poncirin or isomer | AFI | No | No |
| 7.12 | 771.23566 | 771.23531 | -0.45 | [M-H]^−^ | 771.2400; 161.0240 | C_34_H_44_O_20_ | Alhagidin | MOC | No | No |
| 7.13 | 597.18121 | 597.18195 | 1.23 | [M+H]^+^ | 289.0705;153.0282; 163.0389;85.0289 | C_27_H_32_O_15_ | Neoeriocitrin or isomer | AFI | No | No |
| 7.15 | 289.07065 | 289.07066 | 0.03 | [M+H]^+^ | 271.0604;163.0389;153.0182 | C_15_H_12_O_6_ | Eriodictyol | AFI | Yes | Yes |
| 7.18 | 649.24950 | 649.25018 | 1.05 | [M-H]^−^ | 605.2568; 443.2086; 347.1879; 227.1439; 165.0911 | C_32_H_42_O_14_ | Limonin glucoside | AFI | No | No |
| 7.19 | 471.20175 | 471.20134 | -0.87 | [M+H]^+^ | 425.1966; 213.0906; 161.0597 | C_26_H_30_O_8_ | Limonin | AFI | No | No |
| 7.25 | 193.04962 | 193.04953 | -0.47 | [M+H]^+^ | 133.0648; 149.0234; 165.5441; 178.0258 | C_10_H_8_O_4_ | Scopoletin | AFI | Yes | Yes |
| 7.26 | 623.19891 | 623.19814 | -1.23 | [M-H]^−^ | 461.161;315.1097 | C_29_H_36_O_15_ | Magnoloside A | MOC | No | No |
| 7.33 | 625.17816 | 625.17741 | -1.20 | [M-H]^−^ | 317.0668;161.0238; 125.0233 | C_28_H_34_O_16_ | 2-(2,5-Dihydroxy-4-methoxyphenyl)- 5-hydroxy-4-oxo-3,4-dihydro-2Hchromen-7-yl 6-O-(6-deoxy-alpha-Lmannopyranosyl)-β-Dglucopyranoside or isomer | AFI | No | No |
| 7.38 | 515.11963 | 515.11950 | -0.25 | [M-H]^−^ | 191.0556;179.0343; 173.0449; 135.0442; | C_25_H_24_O_12_ | 3,5-Dicaffeoylquinic acid | GF | No | No |
| 7.41 | 577.15698 | 577.15627 | -1.23 | [M-H]^−^ | 269.0457 | C_27_H_30_O_14_ | Rhoifolin | AFI | No | No |
| 7.52 | 356.18536 | 356.18563 | 0.77 | [M+H]^+^ | 311.1273; 296.1039; 280.1090; 265.0857;253.0859 | C_21_H_25_NO_4_ | Glaucine | MOC | Yes | Yes |
| 7.62 | 579.17096 | 579.17193 | 1.67 | [M-H]^−^ | 271.0615;151.0028 | C_27_H_32_O_14_ | Narirutin | AFI | Yes | No |
| 7.63 | 581.18561 | 581.18648 | 1.50 | [M+H]^+^ | 273.0754;195.0288; 85.0289 | C_27_H_32_O_14_ | Naringin | AFI | No | No |
| 7.64 | 273.07529 | 273.07575 | 1.68 | [M+H]^+^ | 255.0656; 231.0643; 153.01816; 147.04399; 119.0493 | C_15_H_12_O_5_ | Naringenin | AFI | Yes | Yes |
| 7.70 | 301.03558 | 301.03537 | -0.70 | [M-H]^−^ | 151.0027; 196.0008; 134.0363; 107.0127; 83.0126 | C_15_H_10_O_7_ | Quercetin | GF | No | No |
| 7.71 | 515.11945 | 515.11950 | 0.10 | [M-H]^−^ | 353.0881;191.0556;179.0343;173.0449; 135.0442 | C_25_H_24_O_12_ | 4,5-Dicaffeoyl quinic acid | GF | No | No |
| 7.72 | 611.19617 | 611.19704 | 1.42 | [M+H]^+^ | 303.0859;195.0287;153.0181;85.0289 | C_28_H_34_O_15_ | Hesperidin | AFI | Yes | Yes |
| 7.74 | 609.18219 | 609.18249 | 0.50 | [M-H]^−^ | 343.0815;301.0718; 151.0028 | C_28_H_34_O_15_ | Hesperidin | AFI | No | No |
| 7.75 | 271.06113 | 271.06120 | 0.24 | [M-H]^−^ | 227.0713; 177.0186; 151.0028; 119.0491 | C_15_H_12_O_5_ | Naringenin or isomer | AFI | No | No |
| 7.78 | 433.11401 | 433.11402 | 0.02 | [M-H]^−^ | 271.0615; 151.0029; 119.0492 | C_21_H_22_O_10_ | Naringenin-7-O-glucoside | AFI | Yes | Yes |
| 7.82 | 659.16217 | 659.16176 | -0.63 | [M-H]^−^ | 497.1307; 435.1279; 335.0754; 191.0555; 161.0447 | C_31_H_32_O_16_ | 3,5-di-O-Caffeoyl-4- O-(3-hydroxy3-methyl)- glutaroylquinic acid | GF | No | No |
| 7.91 | 449.14352 | 449.14422 | 1.56 | [M+H]^+^ | 263.0548;195.0288; 177.0545 | C_22_H_24_O_10_ | poncirenin | AFI | Yes | Yes |
| 7.94 | 465.13867 | 465.13913 | 0.99 | [M+H]^+^ | 303.0863; 177.0545;153.0181 | C_22_H_24_O_11_ | Quercimeritrin | AFI | Yes | Yes |
| 7.99 | 859.32495 | 859.32413 | -0.95 | [M+HCOO]^-^ | 327.1607; 179.0561;89.0232 | C_38_H_54_O_19_ | Crocin II | GF | No | No |
| 7.99 | 837.31549 | 837.31515 | -0.41 | [M+H]^+^ | 675.2622; 513.2107; 351.1563; 347.0945 | C_38_H_54_O_19_ | Crocin II | GF | No | No |
| 8.05 | 551.21399 | 551.21340 | -1.07 | [M-H]^−^ | 521.2057;205.0502 | C_27_H_36_O_12_ | 6'-O-trans-sinapoyl jasminoside L | GF | No | No |
| 8.07 | 179.03394 | 179.03388 | -0.34 | [M+H]^+^ | 179.0702;161.0596; 155.9328; 151.0756 | C_9_H_6_O_4_ | 5,7-Dihydroxycoumarin | AFI | No | No |
| 8.08 | 463.09042 | 463.08819 | -4.82 | [M-H]^−^ | 301.0718; 151.0028 | C_21_H_20_O_12_ | hyperoside | GF | No | No |
| 8.09 | 303.08621 | 303.08631 | 0.33 | [M+H]^+^ | 285.0755; 153.0181 | C_16_H_14_O_6_ | Hesperitin | AFI | No | No |
| 8.10 | 287.09128 | 287.09140 | 0.42 | [M+H]^+^ | 269.0862; 241.5201; 161.0596; 153.0181; 133.0648 | C_16_H_14_O_5_ | Dihydroxy-monomethoxyflavanone | AFI | No | No |
| 8.42 | 315.12430 | 315.12379 | -1.62 | [M-H]^−^ | 267.1028;249.0921;221.0968 | C_18_H_20_O_5_ | Magnolignan B | MOC | Yes | No |
| 8.45 | 261.11200 | 261.11213 | 0.50 | [M+H]^+^ | 243.1014; 189.05449; 159.04399; 131.04916; 103.0545 | C_15_H_16_O_4_ | Suberenol | AFI | Yes | Yes |
| 8.46 | 455.20624 | 455.20642 | 0.40 | [M+H]^+^ | 409.2009;391.1899;161.0597;133.0648; 95.0132 | C_26_H_30_O_7_ | Obacunone | AFI | No | No |
| 8.47 | 633.25555 | 633.25526 | -0.45 | [M-H]^−^ | 427.2129; 359.1861; 331.1917; 205.1226; 101.0232 | C_32_H_42_O_13_ | Obacunone glucoside | MOC | No | No |
| 9.00 | 593.18744 | 593.18758 | 0.23 | [M-H]^−^ | 285.0769;151.0028 | C_28_H_34_O_14_ | Neoponcirin | AFI | Yes | No |
| 9.30 | 728.39758 | 728.39775 | 0.23 | [M+H]^+^ | 700.4060; 615.3132; 587.3181; 502.2283; 474.2338; 377.1817; 339.1664 | C_36_H_53_N_7_O_9_ | Citrusin Ⅲ | AFI | No | No |
| 9.41 | 975.37158 | 975.37148 | -0.11 | [M-H]^−^ | 327.1603; 179.0550 | C_44_H_64_O_24_ | Crocin I | GF | No | No |
| 9.48 | 329.17440 | 329.17473 | 1.00 | [M+H]^+^ | 178.0773; 177.0908; 165.0698; 141.0695;128.0620 | C_20_H_24_O_4_ | Crocetin | GF | No | No |
| 9.61 | 271.05978 | 271.06009 | 1.14 | [M+H]^+^ | 271.0597; 153.0179 | C_15_H_10_O_5_ | Apigenin | AFI | No | No |
| 9.65 | 299.12872 | 299.12888 | 0.53 | [M-H]^−^ | 281.1184;239.1077; 221.0964; 133.0649; 93.0333 | C_18_H_20_O_4_ | Magnolignan A | MOC | Yes | No |
| 9.98 | 281.11829 | 281.11832 | 0.10 | [M-H]^−^ | 263.1079; 133.0648 | C_18_H_18_O_3_ | magnolignan D | MOC | No | No |
| 10.02 | 297.11325 | 297.11323 | -0.06 | [M-H]^−^ | 267.1028; 249.0921; 225.0918; | C_18_H_18_O_4_ | Magnolignan E | MOC | No | No |
| 10.29 | 241.08646 | 241.08702 | 2.31 | [M-H]^−^ | 223.0759;197.0964;95.0126 | C_15_H_14_O_3_ | Randaiol | MOC | No | No |
| 10.30 | 241.08664 | 241.08702 | 1.56 | [M-H]^−^ | 223.0761; 197.0967;157.0655; 133.0651; | C_15_H_14_O_3_ | Magnotriol A | MOC | No | No |
| 11.23 | 253.08691 | 253.08701 | 0.40 | [M-H]^−^ | 235.0762;225.0933; 207.0811 | C_16_H_14_O_3_ | 2,2'-Dihydroxy-5'-(2-propenyl)-1,1'-biphenyl-5-carbaldehyde | MOC | No | No |
| 11.24 | 253.08691 | 253.08701 | 0.40 | [M-H]^−^ | 235.0762; 223.1322; 207.0812 | C_16_H_14_O_3_ | Magnaldehyde E or isomer | MOC | No | No |
| 11.54 | 403.13861 | 403.13984 | 3.05 | [M+H]^+^ | 388.1149; 373.0914; 327.0860; 387.1069 | C_21_H_22_O_8_ | Hexamethoxyflavone | AFI | No | No |
| 11.55 | 403.13812 | 403.13984 | 4.27 | [M+H]^+^ | 373.0911;327.0856 | C_21_H_22_O_8_ | Nobiletin | AFI | No | No |
| 11.61 | 343.11758 | 343.11761 | 0.10 | [M+H]^+^ | 327.0858; 313.0702; 299.0913; 282.0882 | C_19_H_18_O_6_ | Tetramethoxyflavone | AFI | No | No |
| 11.68 | 279.10260 | 279.10266 | 0.21 | [M-H]^−^ | 261.0921;233.0969 | C_18_H_16_O_3_ | Randainal | MOC | Yes | No |
| 11.96 | 433.14951 | 433.14930 | -0.48 | [M+H]^+^ | 418.12540; 403.10202; 385.0916; | C_22_H_24_O_9_ | Hepamethoxylflavone | AFI | No | No |
| 11.98 | 433.14902 | 433.14930 | 0.65 | [M+H]^+^ | 418.1255;403.1018 | C_22_H_24_O_9_ | 3,3',4',5,5',7,8-Heptamethoxyflavone | AFI | No | No |
| 12.15 | 419.13370 | 419.13366 | -0.10 | [M+H]^+^ | 404.1097; 389.0863; | C_21_H_22_O_9_ | Monohydroxy-hexamethoxyflavone | AFI | No | No |
| 12.21 | 419.13306 | 419.13366 | 1.43 | [M+H]^+^ | 404.1093;389.0863;361.0915 | C_21_H_22_O_9_ | 8-Hydroxy-3,5,6,7,3',4'-hexamethoxyflavone | AFI | No | No |
| 12.25 | 373.12796 | 373.12817 | 0.56 | [M+H]^+^ | 358.1039; 343.0806; 315.0847 | C_20_H_20_O_7_ | Pentamethoxyflavone | AFI | Yes | Yes |
| 12.71 | 389.12314 | 389.12309 | -0.12 | [M+H]^+^ | 373.0916; 359.0755; 345.0983; 328.0936 | C_20_H_20_O_8_ | Monohydroxy-pentamethoxyflavone | AFI | No | No |
| 12.75 | 389.12271 | 389.12309 | 0.99 | [M+H]^+^ | 374.0988;359.0753;341.0648 | C_20_H_20_O_8_ | 5-O-Demethylnobiletin | MOC | No | No |
| 13.03 | 203.03320 | 203.03388 | 3.35 | [M+H]^+^ | 175.1482;147.1167 | C_11_H_6_O_4_ | Bergaptol | AFI | Yes | Yes |
| 13.91 | 281.11853 | 281.11832 | -0.76 | [M-H]^−^ | 240.0787; 196.0886; 164.0471; 133.0648; | C_18_H_18_O_3_ | Obovatol | MOC | No | No |
| 14.02 | 265.12323 | 265.12340 | 0.65 | [M-H]^−^ | 247.1127; 223.0760 | C_18_H_18_O_2_ | Magnolol | MOC | No | No |
| 14.03 | 265.12317 | 265.12340 | 0.88 | [M-H]^−^ | 224.0838;223.0752 | C_18_H_18_O_2_ | Honokiol | MOC | No | No |
