# Supplementary material for: Discovery of the Potential Novel Pharmacodynamic Substances From Zhi-Zi-Hou-Po Decoction Based on the Concept of Co-Decoction Reaction and Analysis Strategy
Source: Front Pharmacol. 2022 Jan 13;12:830558. doi: 10.3389/fphar.2021.830558 (PMC8793358; doi:10.3389/fphar.2021.830558)
Supplement: Supplementary file 2 [file Table2.DOCX]

**Table S2.** The MS information of potential new generated compounds from *combined decoction*, and new compounds absorbed into blood and brain.

| **New Compound** | **Rt(min)** | **Average Mz** | **Adduct type** | **MS/MS assigned** | **MS/MS** | **Blood** | **Brain** |
| --- | --- | --- | --- | --- | --- | --- | --- |
| New 1 | 0.99 | 600.86359 | [M-H]^-^ | TRUE | - | No | No |
| New 2 | 1.07 | 294.84256 | [M+H]^+^ | TRUE | 277.1036; 259.0921; 241.0822;211.0710; 194.0447; 165.0656;133.0607; 87.0557 | No | No |
| New 3 | 1.29 | 275.12396 | [M+H]^+^ | TRUE | - | No | No |
| New 4 | 1.30 | 347.02383 | [M-H]^-^ | TRUE | 347.0426; 290.9341; 262.9405; 248.9260; 220.9278; 191.0193; 133.0133; 115.0024; 85.0281 | No | No |
| New 5 | 1.31 | 273.19205 | [M+H]^+^ | TRUE | 273.1440; 249.0274; 219.0172; 181.5410; 144.1018; 130.0975; 112.0872 | No | No |
| New 6 | 1.32 | 274.02573 | [M-H]^-^ | TRUE | 274.0251;257.0108; 232.9221; 159.0097; 115.0026; 96.9589; 71.0126 | No | No |
| New 7 | 1.33 | 466.17130 | [M+H]^+^ | TRUE | - | No | No |
| New 8 | 1.38 | 139.99797 | [M-H]^-^ | TRUE | 140.9819;112.9870; 96.0079; 68.9969 | No | No |
| New 9 | 1.49 | 447.95914 | [M-H]^-^ | TRUE | - | No | No |
| New 10 | 1.52 | 342.04440 | [M-H]^-^ | TRUE | 296.0303; 282.0843; 251.9065; 207.9122; 191.0190; 150.0412; 111.0076 | No | No |
| New 11 | 1.54 | 253.09311 | [M-H]^-^ | TRUE | 240.5363; 217.8499; 210.3488; 184.9039; 156.9099; 133.0131; 115.0025; 96.9588; 71.0127 | No | No |
| New 12 | 1.72 | 188.05579 | [M+H]^+^ | TRUE | 188.0706;170.0600; 160.0967; 142.0499; 128.0705; 84.0449; 56.9656 | No | No |
| New 13 | 1.74 | 275.00735 | [M+H]^+^ | TRUE | 275.1097; 226.8932; 216.0178; 193.0016; 174.9913; 151.0387; 128.9863; 98.9756; 84.9601 | No | No |
| New 14 | 1.77 | 297.11932 | [M-H]^-^ | TRUE | 297.2435; 229.00740; 215.0344; 194.9264; 166.9231; 154.4900; 138.9280; 121.4370; 96.9586; 78.9578 | No | No |
| New 15 | 1.90 | 380.09457 | [M]^+^ | TRUE | 380.0946; 311.4019; 218.0424; 200.0316; 156.5121; 139.0388; 70.0656 | No | No |
| New 16 | 2.07 | 371.09906 | [M-H]^-^ | TRUE | 274.8468; 233.2601; 209.0453; 191.0555; 166.9220; 135.0439 | No | No |
| New 17 | 2.09 | 240.12343 | [M+H]^+^ | TRUE | 240.1205; 236.2373; 222.1125; 204.1014; 167.0701; 162.0911; 140.0342; 126.0914; 112.0758; 86.0605 | No | No |
| New 18 | 2.97 | 365.06476 | [M-H]^-^ | TRUE | 355.9522; 312.0094; 272.7685; 252.5820; 229.3333; 167.0341; 152.0106; 123.0441;68.0920 | No | No |
| New 19 | 3.06 | 406.20825 | [M+H]^+^ | TRUE | 382.7530; 375.4619; 310.0465; 230.1845; 206.9218; 181.3574; 146.3102; 98.4362; 76.0756 | No | No |
| New 20 | 3.34 | 343.10416 | [M-H]^-^ | TRUE | 299.5003; 262.4969; 229.6681; 191.1099; 181.0502; 162.8385; 137.0601; 93.0334 | No | No |
| New 21 | 3.57 | 351.08591 | [M-H]^-^ | TRUE | 315.1105; 209.7378; 178.7464; 135.0441; 119.0337; 101.0232; 89.0232; 59.0125 | No | No |
| New 22 | 3.89 | 537.18250 | [M-H]^-^ | TRUE | 473.7290; 407.9136; 293.0872; 273.0498; 233.0666; 197.0818; 99.0076; 73.0281 | No | No |
| New 23 | 4.17 | 353.14609 | [M-H]^-^ | TRUE | 353.1473;334.8227; 272.8747; 250.8865; 221.1027; 161.0449; 101.0233; 71.0126 | No | No |
| New 24 | 4.19 | 216.10194 | [M+H]^+^ | TRUE | 216.1228; 192.9473; 174.9578; 151.9399; 121.0650; 96.0448; 84.9602;71.0498 | Yes | No |
| New 25 | 4.30 | 647.21539 | [M]^+^ | TRUE | 647.2169; 501.1561; 299.6801; 139.9626; 111.2210; 65.2638 | No | No |
| New 26 | 4.30 | 435.15161 | [M-H]^-^ | TRUE | 398.2542; 353.1427; 174.9554; 146.9604; 102.0559; 56.9093 | No | No |
| New 27 | 4.30 | 721.18719 | [M-H]^-^ | TRUE | 671.0301; 544.8124; 477.1639; 135.0438; 96.9589 | No | No |
| New 28 | 4.38 | 297.10950 | [M-H]^-^ | TRUE | 297.2434;260.8818; 229.2941; 180.91211; 166.9231; 151.0505; 138.9281; 108.0443;78.9577 | No | No |
| New 29 | 4.52 | 455.15320 | [M+H]^+^ | TRUE | 455.1518; 316.5020; 187.1474; 163.0385; 105.0338; 62.3013 | No | No |
| New 30 | 4.56 | 153.86749 | [M-H]^-^ | TRUE | 153.8678; 126.9025; 96.9590; 78.9578; 61.9871 | No | No |
| New 31 | 4.56 | 351.07184 | [M-H]^-^ | TRUE | - | No | No |
| New 32 | 4.66 | 505.16779 | [M+H]^+^ | TRUE | 505.1673; 181.0497; 175.0364; 146.8475; 115.3325; 74.8469 | No | No |
| New 33 | 4.71 | 510.14630 | [M-H]^-^ | TRUE | 447.15155;297.1387; 179.0717; 135.0445; 99.0070; 61.9872 | No | No |
| New 34 | 4.72 | 487.18045 | [M+H]^+^ | TRUE | 487.1788; 407.5055;299.8138; 259.0791;250.0807; 205.0472; 163.0389; 146.8855;121.2603;74.8434 | No | No |
| New 35 | 4.77 | 367.10083 | [M+H]^+^ | TRUE | - | No | No |
| New 36 | 4.82 | 450.19745 | [M+H]^+^ | TRUE | - | No | No |
| New 37 | 4.82 | 565.96259 | [M-H]^-^ | TRUE | - | No | No |
| New 38 | 4.82 | 527.98859 | [M-H]^-^ | TRUE | - | No | No |
| New 39 | 4.82 | 197.09222 | [M+H]^+^ | TRUE | - | No | No |
| New 40 | 4.82 | 563.96637 | [M-H]^-^ | TRUE | - | No | No |
| New 41 | 4.95 | 499.14294 | [M+H]^+^ | TRUE | 499.1417; 408.4180; 367.1005; 337.9048; 229.5044; 175.5489; 147.0156; 11.6254; 68.6636 | No | No |
| New 42 | 4.98 | 422.13086 | [M-H]^-^ | TRUE | 281.5440; 141.3093; 61.9871 | No | No |
| New 43 | 4.99 | 367.10074 | [M+H]^+^ | TRUE | 367.0999; 205.0474; 185.0427; 148.2006; 85.0290; 57.4273 | No | No |
| New 44 | 5.03 | 466.11984 | [M-H]^-^ | TRUE | 4040.0129; 368.2878; 286.7159; 180.2039; 61.9871 | No | No |
| New 45 | 5.08 | 501.91208 | [M-H]^-^ | TRUE | 477.2608; 299.5777; 193.0144; 162.8380; 146.9458; 96.9589 | No | No |
| New 46 | 5.17 | 237.12369 | [M+H]^+^ | TRUE | 236.9865; 213.9701;190.9526; 172.9544; 154.9422; 120.0809; 115.9642;56.9655 | Yes | No |
| New 47 | 5.25 | 554.17163 | [M-H]^-^ | TRUE | 522.4724; 456.6928; 363.0397; 333.1362; 61.9871 | No | No |
| New 48 | 5.29 | 379.10040 | [M+H]^+^ | TRUE | 379.1003; 299.4806; 229.7255; 216.0876; 203.3941; 176.0203; 148.7733; 121.4865; 85.0288; 74.4084 | No | No |
| New 49 | 5.35 | 381.09476 | [M+H]^+^ | TRUE | 218.0444; 146.9961; 121.4767;105.0338; 74.8718 | No | No |
| New 50 | 5.40 | 271.11487 | [M+H]^+^ | TRUE | 271.1153; 258.0286; 240.0181; 224.9333; 190.9620; 172.9516; 147.0438; 113.3122; 102.9708; 84.9602 | No | No |
| New 51 | 5.42 | 351.10516 | [M+H]^+^ | TRUE | 351.1046; 297.7427; 271.1275; 229.6710; 104.5742; 85.0288; 56.9654 | No | No |
| New 52 | 5.45 | 337.09363 | [M-H]^-^ | TRUE | 238.8782; 213.7103;191.0555; 163.0393; 119.0492;96.9589; 85.0281 | No | No |
| New 53 | 5.50 | 507.99454 | [M-H]^-^ | TRUE | 472.0220; 434.0421;299.7081; 229.0591; 184.8865; 144.8726;61.9871 | No | No |
| New 54 | 5.50 | 505.99719 | [M-H]^-^ | TRUE | 470.0204; 434.0442; 266.9379; 242.9889;184.8860; 157.8621; 142.8750; 125.8721; 103.9190; 96.9590 | No | No |
| New 55 | 5.57 | 377.08499 | [M+H]^+^ | TRUE | 377.0837; 359.0727; 331.0798;299.9338; 215.0527; 197.0417; 163.0389;137.0599; 105.0340; 69.0340 | No | Yes |
| New 56 | 5.73 | 389.06488 | [M-H]^-^ | TRUE | 299.6303; 229.5774; 191.0344; 181.0497; 166.02652; 121.4028; 91.7974 | No | No |
| New 57 | 5.77 | 397.08987 | [M]^+^ | TRUE | 397.1464;234.0860; 227.0172; 185.0416; 163.0393; 139.0390;108.0447; 85.0289;58.0659 | No | No |
| New 58 | 5.95 | 411.12506 | [M+H]^+^ | TRUE | 411.1258; 249.0732; 203.0519; 163.0391;137.0599; 105.0337; 85.0290 | No | No |
| New 59 | 6.10 | 379.10013 | [M+H]^+^ | TRUE | - | No | No |
| New 60 | 6.14 | 448.10925 | [M-H]^-^ | TRUE | 407.9566; 274.9572; 205.0505; 152.9173; 61.9871 | No | No |
| New 61 | 6.25 | 511.14059 | [M+H]^+^ | TRUE | - | No | No |
| New 62 | 6.26 | 336.14285 | [M+H]^+^ | TRUE | - | No | No |
| New 63 | 6.36 | 508.12775 | [M-H]^-^ | TRUE | 425.0378; 354.2621; 325.1341; 249.0488; 166.9236; 131.0341; 89.0232; 61.9871 | No | No |
| New 64 | 6.38 | 273.06140 | [M-H]^-^ | TRUE | 273.0404; 244.7695; 229.0450; 185.0607; 167.0499; 130.9826; 85.0283 | No | No |
| New 65 | 6.47 | 501.19553 | [M+H]^+^ | TRUE | 501.1937; 486.1688; 407.9503; 339.1412; 317.0838; 283.5325; 237.0282; 177.0546; 145.0283 | No | No |
| New 66 | 6.48 | 408.15213 | [M-H]^-^ | TRUE | 368.5609; 340.1555; 325.1319; 310.1083; 125.0234 | No | No |
| New 67 | 6.48 | 355.10376 | [M-H]^-^ | TRUE | - | No | No |
| New 68 | 6.65 | 543.24060 | [M+H]^+^ | TRUE | - | No | No |
| New 69 | 6.65 | 683.32556 | [M+H]^+^ | TRUE | 670.9372; 587.2941; 407.8726; 353.1570; 246.9960; 163.0392; 85.0288 | No | No |
| New 70 | 6.66 | 491.99893 | [M-H]^-^ | TRUE | 455.0309; 407.9717; 329.0894; 233.0660; 151.0392; 89.0233 | No | No |
| New 71 | 6.73 | 650.24402 | [M+H]^+^ | TRUE | - | No | No |
| New 72 | 6.81 | 251.10287 | [M+H]^+^ | TRUE | - | No | No |
| New 73 | 6.85 | 497.16284 | [M+H]^+^ | TRUE | 497.1596; 439.1560; 408.2204; 383.2116; 325.0591; 237.0282; 185.0401; 163.0388; 121.4797; 85.0290 | No | No |
| New 74 | 6.90 | 367.10419 | [M-H]^-^ | TRUE | 282.6946; 232.7845;179.5638;159.8592;146.5573;102.0644;86.9580 | No | No |
| New 75 | 7.00 | 542.15259 | [M-H]^-^ | TRUE | 408.3545; 229.4809; 161.0237; 132.4659; 61.9871 | No | No |
| New 76 | 7.19 | 512.10535 | [M-H]^-^ | TRUE | 299.3024;287.0559; 191.0556; 151.0028; 123.0441; 61.9871 | No | No |
| New 77 | 7.20 | 391.16217 | [M-H]^-^ | TRUE | - | No | No |
| New 78 | 7.22 | 492.20856 | [M-H]^-^ | TRUE | 295.1180; 274.5122; 149.5060; 126.0661; 61.9872 | No | No |
| New 79 | 7.41 | 421.12692 | [M-H]^-^ | TRUE | - | No | No |
| New 80 | 7.41 | 523.17883 | [M+H]^+^ | TRUE | 523.1810; 461.1783; 417.1313; 379.1359; 298.1083; 181.0495; 163.0389;58.0659 | No | No |
| New 81 | 7.46 | 347.17102 | [M-H]^-^ | TRUE | - | No | No |
| New 82 | 7.47 | 481.16803 | [M+H]^+^ | TRUE | - | No | No |
| New 83 | 7.48 | 638.19403 | [M-H]^-^ | TRUE | - | No | No |
| New 84 | 7.53 | 363.12100 | [M-H]^-^ | TRUE | - | No | No |
| New 85 | 7.63 | 292.11731 | [M]^+^ | TRUE | - | No | No |
| New 86 | 7.64 | 564.22925 | [M-H]^-^ | TRUE | 426.4651;407.7779; 240.2599; 136.7444; 61.9872 | No | No |
| New 87 | 7.69 | 454.09998 | [M-H]^-^ | TRUE | 426.4651;407.7779; 240.2599; 136.7444; 61.9872 | No | No |
| New 88 | 7.72 | 564.23132 | [M-H]^-^ | TRUE | 407.9018; 382.9611; 205.0502; 223.0605; 190.0268; 61.9871 | No | No |
| New 89 | 7.72 | 313.12512 | [M]^+^ | TRUE | - | No | No |
| New 90 | 7.74 | 526.21454 | [M-H]^-^ | TRUE | 508.3396; 477.6120; 216.3790; 165.0918;136.1626; 61.9871 | No | No |
| New 91 | 7.74 | 1305.40601 | [M-H]^-^ | TRUE | 917.8721; 780.24500; 609.1305; 301.0719; 225.0765;123.0442 | No | No |
| New 92 | 7.85 | 374.10895 | [M-H]^-^ | TRUE | - | No | No |
| New 93 | 7.88 | 485.17722 | [M+H]^+^ | TRUE | - | No | No |
| New 94 | 7.92 | 771.28247 | [M+H]^+^ | TRUE | - | No | No |
| New 95 | 7.93 | 785.28979 | [M-H]^-^ | TRUE | 609.1798; 506.7528; 408.3679; 257.5456; 175.0969;72.9918 | No | No |
| New 96 | 7.94 | 541.22534 | [M+H]^+^ | TRUE | - | No | No |
| New 97 | 7.94 | 527.15063 | [M+H]^+^ | TRUE | 527.2458; 473.8106; 395.2030; 317.0832; 275.0741; 229.2942; 137.0594 | No | No |
| New 98 | 7.96 | 520.16791 | [M-H]^-^ | TRUE | 367.2960; 231.9186; 205.0506; 95.2462; 61.9871 | No | No |
| New 99 | 7.99 | 837.31268 | [M+H]^+^ | TRUE | 675.2622; 583.2048; 513.2107; 347.0945; 259.0755; 185.0424 | No | No |
| New 100 | 7.99 | 876.31818 | [M-H]^-^ | TRUE | 735.7362; 671.02142; 586.9719; 96.9588 | No | No |
| New 101 | 8.03 | 537.23169 | [M+H]^+^ | TRUE | 537.2304;502.7914; 375.1769; 367.0997; 307.0783; 207.0643; 175.0391; 137.0594; 119.0491; 85.0289 | No | No |
| New 102 | 8.19 | 499.15848 | [M+H]^+^ | TRUE | 499.1572;457.1471; 343.1042; 302.0766; 237.0284; 193.0490; 163.0387; 137.0596; 85.0288 | No | No |
| New 103 | 8.33 | 501.15393 | [M]^+^ | TRUE | 487.9677;407.7604; 312.0854; 235.1697; 217.1588; 207.1377; 163.0387; 137.0595; 121.4699 | No | No |
| New 104 | 8.38 | 587.15186 | [M]^+^ | TRUE | 587.1541; 407.9043; 230.1250; 167.0697; 126.0698; 126.6498; 102.4420;85.0289 | No | No |
| New 105 | 8.43 | 524.19977 | [M-H]^-^ | TRUE | 400.8402;351.7582; 299.7706; 164.83511; 61.9871 | No | No |
| New 106 | 8.46 | 544.23248 | [M+H]^+^ | TRUE | 408.2835; 335.0805; 266.1176; 249.0908; 219.0804; 191.0853; 145.0279;117.0337 | No | No |
| New 107 | 8.57 | 439.23080 | [M+H]^+^ | TRUE | 439.2294;369.1863; 277.1759; 259.1683;146.8773;103.5103;85.0285 | No | No |
| New 108 | 8.80 | 323.14642 | [M+H]^+^ | TRUE | 323.1456;308.1032;291.1018; 263.1067; 231.0780; 203.0849; 161.0598; 135.0440; 105.0338 | No | No |
| New 109 | 8.83 | 459.16275 | [M+H]^+^ | TRUE | 459.1619;380.0245; 357.4745; 299.9115; 234.0861; 205.0469; 176.0449; 105.0337 | No | No |
| New 110 | 8.91 | 277.10727 | [M+H]^+^ | TRUE | - | No | No |
| New 111 | 8.92 | 301.03491 | [M-H]^-^ | TRUE | 301.0358; 254.8568; 178.9981; 164.9255;151.0028;136.9310; 121.0283 | No | No |
| New 112 | 8.97 | 609.23163 | [M+H]^+^ | TRUE | - | No | No |
| New 113 | 8.98 | 296.12762 | [M+H]^+^ | TRUE | - | No | No |
| New 114 | 8.98 | 681.21283 | [M+H]^+^ | TRUE | 651.2022;621.2015; 456.1384; 441.1154; 303.0836;248.0657; 230.0547; 131.0491;85.0288 | No | No |
| New 115 | 8.99 | 719.19409 | [M]^+^ | TRUE | 719.18011; 588.1002;457.2220; 413.0833;397.0524; 329.0851; 316.9588; 185.0415; 135.1002;74.9220 | No | No |
| New 116 | 9.02 | 609.22882 | [M+H]^+^ | TRUE | - | No | No |
| New 117 | 10.61 | 418.22299 | [M+H]^+^ | TRUE | 389.0864; 299.8020; 211.0226; 203.0337; 153.1273; 135.1169; 105.0337; 81.0704; 71.0497 | No | No |
| New 118 | 10.73 | 987.35175 | [M-H]^-^ | TRUE | 918.5323;735.7524;501.2170;327.1602;283.1700. 173.0448 | No | No |
| New 119 | 11.02 | 293.15381 | [M+H]^+^ | TRUE | 293.2116; 275.2009; 247.0832; 223.1325; 181.1217;163.1117; 145.1011;121.1016; 107.0859; 95.0495; 81.0704; 67.0550;55.0550 | No | No |
| New 120 | 11.02 | 687.24109 | [M-H]^-^ | TRUE | 557.0247; 509.6982; 352.4706; 283.1704; 239.1800; 161.0452 | No | No |
| New 121 | 11.02 | 675.26257 | [M]^+^ | TRUE | 675.2551;587.4324;486.8657; 365.1043;347.0945; 300.1052;259.0789;203.0526; 169.0348; 103.4908 | No | No |
| New 122 | 11.54 | 651.26703 | [M-H]^-^ | TRUE | 544.5344;299.4949; 283.1717; 239.1811; 119.0339;89;0232; 59.0126; | No | No |
| New 123 | 12.20 | 308.12775 | [M+H]^+^ | TRUE | 308.2964;299.8317;249.0907;238.9164;219.0803;210.9217;191.0854; 174.9003;121.4558; 86.0605;71.0865 | No | No |
| New 124 | 14.39 | 293.11887 | [M-H]^-^ | TRUE | 293.1186; 265.1238; 273.0924; 247.1125; 229.99603; 205.9012; 166.0615;96.9590 | No | No |
| New 125 | 16.68 | 529.23846 | [M-H]^-^ | TRUE | 529.2398; 511.2285; 470.1879; 299.7023; 298.8664; 198.8664; 59.5248 | No | No |
